# Deciphering the Potential Role of Specialized Pro-Resolving Mediators in Obesity-Associated Metabolic Disorders

**DOI:** 10.3390/ijms25179598

**Published:** 2024-09-04

**Authors:** Nahyun Kim, Ha Youn Shin

**Affiliations:** Department of Biomedical Science and Engineering, Konkuk University, Seoul 05029, Republic of Korea; knh64@naver.com

**Keywords:** obesity, metabolic disorders, specialized pro-resolving mediators

## Abstract

Obesity-related metabolic disorders, including diabetes, non-alcoholic fatty liver disease (NAFLD), and cardiovascular disease, increasingly threaten global health. Uncontrolled inflammation is a key pathophysiological factor in many of these conditions. In the human body, inflammatory responses generate specialized pro-resolving mediators (SPMs), which are crucial for resolving inflammation and restoring tissue balance. SPMs derived from omega-3 polyunsaturated fatty acids (n-3 PUFAs) such as resolvins, protectins, and maresins hold promise in attenuating the chronic inflammatory diseases associated with lipid metabolism disorders. Recent research has highlighted the therapeutic potential of n-3 PUFA-derived metabolites in addressing these metabolic disorders. However, the understanding of the pharmacological aspects of SPMs, particularly in obesity-related metabolic disorders, remains limited. This review comprehensively summarizes recent advances in understanding the role of SPMs in resolving metabolic disorders, based on studies in animal models and humans. These studies indicate that SPMs have potential as therapeutic targets for combating obesity, as well as offering insights into their mechanisms of action.

## 1. Introduction

**Figure 1:**
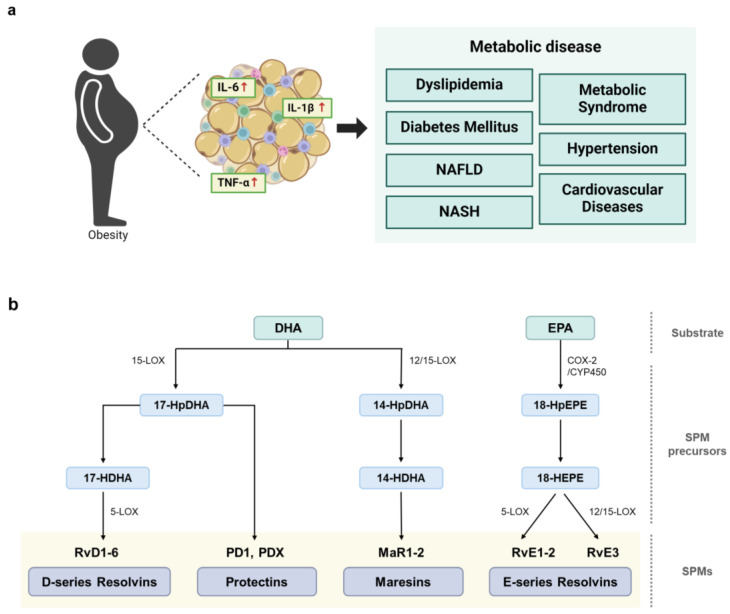
Links between obesity-induced chronic inflammation, metabolic diseases, and the biosynthesis of SPMs to resolve inflammation. (**a**) Obesity induces aberrant inflammatory responses, leading to the development of various types of metabolic diseases. (**b**) Initiation of SPM biosynthesis from long-chain PUFAs, such as DHA and EPA. Enzymes, including lipoxygenase (LOX) and cyclooxygenase (COX), catalyze the conversion of PUFAs into various SPM families. D-series resolvins (RvD1-RvD6), protectins (PD1 and PDX), and maresins (MaR1 and MaR2), are synthesized from DHA. Then, 15-LOX metabolizes DHA to the intermediate molecule 17-HDHA, which is further metabolized to generate D-series resolvins and protectins. Furthermore, 12- or 15-LOX metabolizes DHA to the intermediate molecule 14-HDHA, which is further metabolized to produce maresins. EPA is metabolized by COX-2 or CYP450 to the SPM precursor, 18-hydroxyeicosapentaenoic acid (18-HEPE), which is further metabolized to yield the E-series resolvins RvE1, RvE2, and RvE3.

Obesity is a significant risk factor for various metabolic diseases, including cardiovascular diseases, non-alcoholic fatty liver disease (NAFLD), and diabetes [1,2,3]. Increased rates of obesity are strongly associated with the prevalence of obesity-induced metabolic disorders, posing a major challenge to global public health [4]. Excessive fat accumulation in the human body affects lipid metabolism, leading to high blood pressure, insulin resistance, and chronic inflammation. Because the inability to resolve inflammation is a feature common to obesity-related metabolic diseases [5], persistent inflammation often occurs in various tissues throughout the body, contributing to the progression of metabolic disorders. In particular, chronic low-grade inflammation in adipose tissue has been recognized as a key factor in the development of obesity-associated complications, and further systemic inflammation exacerbates metabolic dysfunction (Figure 1a) [6,7].

The triggering of an inflammatory response results in the metabolism of essential fatty acids and the release of a superfamily of cell signaling molecules called specialized pro-resolving mediators (SPMs) to suppress inflammation. The SPM family includes a diverse array of bioactive lipid molecules crucial for resolving inflammation [8]. Specifically, SPMs consist of chemically and functionally distinct anti-inflammatory and pro-resolving lipid mediators derived from omega-3 (n-3) and omega-6 (n-6) polyunsaturated fatty acids (PUFAs). SPMs include resolvins, protectins, and maresins enzymatically synthesized from n-3 PUFAs such as eicosapentaenoic acid (EPA) and docosahexaenoic acid (DHA), and lipoxins synthesized from the n-6 PUFA arachidonic acid (Figure 1b) [8]. These potent lipid signaling molecules are synthesized during the transition from inflammation to resolution and play crucial roles in restoring the homeostasis of damaged tissue. Indeed, SPMs have dual functions—they both suppress inflammation and activate the resolution process. Collectively, these natural lipid metabolites play essential roles in orchestrating the resolution of inflammation by limiting pro-inflammatory responses and promoting tissue repair through the stimulation of wound healing processes [9,10].

Dysregulation of the SPM biosynthetic pathways in adipose, liver, and other tissues has been shown to result in the progression of lipid metabolic diseases [5]. Specifically, the aberrant regulation of SPM synthesis leads to an imbalance in the synthesis of pro-resolving SPMs, reducing SPM levels in various tissues. These abnormally low SPM levels affect anti-inflammatory responses and metabolic functions in the human body [5,11,12]. Several experimental strategies involving animal models of obesity have been designed to address impaired SPM formation. One notable approach involves boosting SPM levels in the body through dietary intake or injection of supplemental SPMs. For example, the effects of dietary supplementation with the protectin D1 (PD1) precursor 17-HDHA have been assessed in mice with genetically mutated or diet-induced obesity. The levels of PD1 and 17-HDHA in adipose tissue were shown to be lower in the obese mice than in the control mice. Supplementation with 17-HDHA was found to alleviate inflammation in the adipose tissues and to promote insulin sensitivity in mice fed a high-fat diet (HFD) [13]. In addition, supplementation with SPMs was shown to mitigate key features of obesity, such as reducing body weight and hepatic steatosis [14,15]. Furthermore, the benefits of n-3 PUFAs supplementation are extensive and go beyond merely increasing SPM levels. n-3 PUFAs have been shown to improve cardiovascular health, enhance metabolic regulation, and strengthen immune function, thereby contributing to the overall management of metabolic disorders [16,17]. These findings highlight the potential of SPMs to regulate lipid metabolic disorders, such as obesity-induced insulin resistance, through various biological pathways [13,14,15,18].

Although the anti-inflammatory effects of SPMs have been well studied, their therapeutic effects on obesity-associated metabolic disorders have not yet been comprehensively analyzed. This review summarizes key research findings on the effects of supplementing n-3 PUFA-derived SPMs in obesity-induced metabolic disorders, including diabetes, NAFLD, and atherosclerosis, and explores the potential roles of these SPMs as preventive or therapeutic agents. Specifically, this review will describe the effects of obesity on SPM levels, summarize the findings of in vitro cell and in vivo animal studies demonstrating the role of SPMs in resolving obesity-induced metabolic disorders, and describe the therapeutic potential of SPMs in the clinical treatment of various lipid metabolic diseases. These findings highlight the potential role of n-3 PUFA-derived SPMs in obesity-associated metabolic diseases and provide insights into the development of innovative therapeutic approaches.

## 2. Biosynthesis of N-3 PUFA-Derived SPMs

DHA and EPA are omega-3 fatty acids that serve as precursors for various SPMs, including resolvins, protectins, and maresins. The chemical structure of each SPM is unique, but they all function in promoting the resolution of inflammation. SPM synthesis can be enhanced by specific physiological stimuli or non-specific pathological conditions, and SPMs can be sequentially metabolized by various catalytic enzymes, generating several intermediate molecules (Figure 1b) [19]. Resolvins are anti-inflammatory cytokines derived from DHA and EPA and classified as D-series (RvD) and E-series (RvE) resolvins, respectively [20]. D-series resolvins are primarily synthesized from DHA by 15-lipoxygenase (LOX) and 5-LOX. In the first step, DHA is converted to 17S-hydroxy-4Z,7Z,10Z,13Z,15E,19Z-docosahexaenoic acid (17-HDHA) by 15-LOX. Subsequently, 5-LOX catalyzes 17-HDHA oxygenation, transforming 17-HDHA into an intermediate epoxide, which is subsequently hydrolyzed to produce RvD. To date, six members of the RvD family have been identified (RvD1-6). E-series resolvins are generated from EPA through oxygenation catalyzed by cyclooxygenase-2 (COX-2) or microbial cytochrome P450 (CYP450), resulting in the formation of the intermediate compound 18-hydroxyeicosapentaenoic acid (18-HEPE) [21]. Then, 18-HEPE is either epoxidized by 5-LOX to form RvE1 or converted into RvE2 by peroxidase [22,23]. Additionally, RvE3 is produced from 18-HEPE through the 12-LOX or 15-LOX pathway [22,24].

Similar to RvDs, protectins are synthesized from 17-hydroperoxy-DHA (17-HpDHA) intermediates via 15-LOX [25,26], generating several members of the protectin family, including PD1, the first identified member of this family [27], protectin DX (PDX), and an isomer of protectin/neuroprotectin D1. In addition, maresins are primarily synthesized in M2 macrophages which promote anti-inflammatory responses and directly influence phagocytosis. DHA is oxygenated by either 12-LOX or 15-LOX to form 14-hydroxy-docosahexaenoic acid (14-HDHA), an intermediate molecule subsequently converted to members of the maresin family, including MaR1 and MaR2 [28,29].

## 3. Decreased SPM Levels in Models of Obesity: Insights from Animal and Human Studies

**Figure 2:**
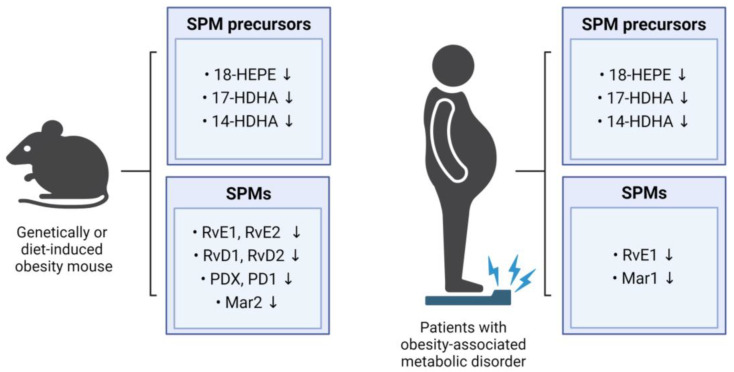
Impact of obesity on SPM levels in mice and humans. The figure summarizes the influence of obesity on SPM levels in mice and humans, showing alterations in the levels of SPM precursors and SPMs.

The chronic low-grade inflammation observed in obesity is due, at least in part, to the impairment of the biological pathways involved in the resolution of inflammation. This persistent inflammatory state facilitates the progression of obesity-related metabolic disorders, such as insulin resistance, type 2 diabetes, atherosclerosis, and NAFLD [30,31,32]. Indeed, several studies have demonstrated reductions in SPM metabolites and their precursors across various tissues in obese mice and humans (Figure 2). These reductions are accompanied in obese individuals by an impairment in the ability of the immune system to effectively resolve the persistent systemic inflammation [33]. SPMs serve as signaling molecules that terminate inflammatory processes by communicating with immune cells. However, under certain conditions, including obesity, the ability to transmit these potent signaling molecules can be diminished, resulting in non-attenuation of inflammation. Overall evidence indicates that SPM levels are negatively associated with obesity, and that reductions in SPM levels may worsen metabolic function, thereby promoting the development of obesity-related metabolic diseases.

### 3.1. Studies on Intra-Body SPM Levels in Animal Models of Obesity

Several studies have revealed that the levels of SPMs and their precursors are lower in the obese mice than in the non-obese mice. For example, the levels of RvD1, RvD2, and PD1 in the adipose tissue were lower in the obese mice than in the lean mice, with RvD1 and RvD2 being specifically identified as the main SPMs reducing inflammatory processes in the adipose tissue [34]. The levels of SPM precursors in the adipose tissues involved in the synthesis of resolvins (18-HEPE), protectins (17-HDHA), and maresins (14-HDHA) were found to be lower in the mice with diet-induced or genetically mutated obesity than in the non-obese mice [13,34]. The levels in the liver and adipose tissues of 18-HEPE, a precursor in resolvin biosynthesis, were also found to be lower in obese than in non-obese mice [11], and the MaR2 levels in the adipose tissues were significantly lower in the aged HFD-induced obese mice than in the young normal controls [35]. In addition, the levels of RvE1, RvE2, RvD1, and RvD2 in the adipose tissue macrophages were lower in the HFD mice than in the lean mice [36]. 

Deficiencies in SPMs are not limited to adipose tissue and liver. For example, the mice fed a Western diet showed significantly lower PDX levels in the spleen [37], and the HFD-fed mice showed lower levels in 14-HDHA, 17-HDHA, and PDX in the spleen and bone marrow [38]. Moreover, lower levels of 17-HDHA, 14-HDHA, and 4-HDHA were observed in dorsal skin wounds from the obese diabetic (db/db) mice with a mutation in the leptin receptor gene than in the wounds of the littermate controls [39]. Levels of RvD2 were reported to be lower in the central nervous system of the obese mice than of the non-obese mice, particularly within the hypothalamus [40], and obesity was found to have a detrimental impact on the synthesis of SPMs and cellular SPM levels across various tissues. Overall, HFD- or genetically induced obese mice consistently exhibited significantly lower concentrations of SPMs or their metabolic intermediates when compared with their lean counterparts. These findings suggest a potential therapeutic avenue for preventing or treating obesity-related conditions by increasing the circulating levels of SPMs and their metabolic intermediates through targeted dietary or pharmacological interventions.

### 3.2. Studies on Intra-Body SPM Levels in Obese Patients

Consistent with findings in mouse studies, human studies confirmed that levels of key SPM precursors and SPMs are lower in the obese individuals than in the lean controls. Specifically, plasma concentrations of 14-HDHA, 17-HDHA, and 18-HEPE were lower in the obese individuals than in the lean ones. Leukocytes from the obese individuals also exhibited significantly lower levels of 17-HDHA and 18-HEPE, along with imbalanced production of resolvins [33]. Weight loss resulted in increased levels of RvE1 in the neutrophils of patients with a metabolic syndrome, providing direct evidence of the influence of obesity on SPM levels [41]. Compared with healthy control individuals, serum RvE1 levels were lower in patients with type 2 diabetes [42]. In addition, serum MaR1 levels were found to be lower in patients with type 2 diabetes and NAFLD than in healthy controls [43,44]. Taken together, these findings suggest that excessive body fat may impair the synthesis of SPMs.

## 4. Effects of SPMs on In Vitro Cell and In Vivo Animal Models of Obesity-Related Metabolic Disorders

Studies in both humans and mice have revealed that obesity significantly reduces SPM levels, suggesting that excess body fat may interfere with the synthesis of SPMs. These reductions, in turn, lead to chronic inflammation and exacerbate obesity-associated metabolic diseases. To address these issues, recent studies tested the effects of SPM supplementation on in vitro cell lines and on in vivo animal models of various obesity-related metabolic diseases. These studies clearly demonstrated that SPM supplementation could alleviate inflammatory responses and ameliorate aberrant lipid metabolisms in both cell lines and animal models. We summarize the effects of supplementation with resolvin, protectin, and maresin, respectively, on obesity-associated metabolic disorders.

### 4.1. Resolvin

#### 4.1.1. D-Series Resolvins

RvD1 plays a significant role in anti-inflammatory activity by promoting a shift in macrophage polarity from the pro-inflammatory M1 phenotype towards the anti-inflammatory M2 phenotype [15,45,46,47]. This phenotype shift inhibits macrophage infiltration into inflamed lesions and resolves inflammation. Indeed, RvD1 supplementation of HFD-fed obese mice induced a phenotype switch from M1 to M2 macrophages and reduced the production of pro-inflammatory cytokines, including TNFα, IL-6, IL-1β, and MCP-1, in inflamed adipose tissue (Table 1) [34,46].

Several studies tested the ability of RvD1 to alleviate the clinical signs of obesity-related metabolic diseases, including NAFLD, more progressive non-alcoholic steatohepatitis (NASH), and diabetes. For example, RvD1 was found to reduce the accumulation of triglycerides (TG) and the expression of the lipid synthesis factor, sterol regulatory element-binding protein 1 (SREBP-1), by inhibiting the c-Jun N-terminal kinase (JNK) pathway in tunicamycin-treated HepG2 cells [14]. Tunicamycin is an antibiotic commonly used to induce stress on the endoplasmic reticulum (ER) of liver cells to develop NAFLD. Pretreatment with RvD1 also prevented ER stress-induced caspase-3-dependent apoptosis. Both ER stress-induced hepatic apoptosis and TG accumulation are key risk factors for the progression of NAFLD [14]. Thus, by lowering TG levels, RvD1 plays a crucial role in inhibiting fat accumulation in the liver [14,15,45].

RvD1 has also been found to enhance insulin sensitivity, resulting in better glucose metabolism [14,15,45]. RvD1 induces the expression of adiponectin, an adipocyte-derived hormone that promotes insulin sensitivity and drives anti-inflammatory activities [15,34,45,46], thereby improving glucose tolerance and reducing insulin and glucose levels [15,45]. Adiponectin enhances insulin sensitivity by activating AMP-activated protein kinase (AMPK) and regulates cell survival and metabolism by modulating protein kinase B (Akt) [48]. AMPK serves as a primary cellular energy sensor and plays a crucial role in regulating metabolic homeostasis [49]. Moreover, RvD1 treatment has been found to increase levels of activated AMPK in the adipose tissues [45] and to enhance insulin sensitivity via Akt phosphorylation, a downstream target of the insulin signaling pathway [45,50], potentially alleviating type 1 and type 2 diabetes in rats [50,51]. These findings suggest that RvD1 upregulates adiponectin, leading to AMPK activation and Akt phosphorylation, subsequently enhancing insulin sensitivity and anti-inflammatory activities. 

RvD2 has also been found to exert anti-inflammatory and pro-resolving effects, which may play a key role in alleviating metabolic disorders. Administration of RvD2 to the inflamed adipose tissue of obese mice restored the impaired expression and secretion of adiponectin while reducing the production of proinflammatory cytokines [34]. Although hypothalamic RvD2 levels were decreased in the HFD-induced obese mice, intra-cerebroventricular injection of RvD2 into the obese mice improved glucose tolerance and mitigated hypothalamic inflammation [40]. RvD2 was also found to be effective in ameliorating atherosclerosis and diabetes-associated acute ischemic stroke in diabetic mice [52,53].

Taken together, these findings showed that RvD1 and RvD2 exhibit potent anti-inflammatory and pro-resolving activities in both in vitro and in vivo models of obesity-associated metabolic diseases, such as NAFLD, NASH, and type 2 diabetes. Resolvin might be a good candidate for treating various obesity-induced metabolic disorders and associated complications.

**Table 1:** Effects of resolvins on in vitro cell lines and in vivo animal models of obesity-induced metabolic diseases.

Metabolite	Target Disease	Experimental Model	Effects	Ref.
RvD1	Obesity	C57BL/6 mice with HFD-induced obesity	Promotes resolution of inflammation in adipose tissue	[46]
		C57BL/6 mice with HFD-induced obesity	Rescues impaired expression and secretion of adiponectin	[34]
	NAFLD	HepG2 cells with tunicamycin treatment	Prevents hepatic apoptosis and lipid accumulation by inhibiting the JNK-dependent pathway	[14]
	NASH	C57BL/6 mice with HFD-induced obesity	Ameliorates hepatic steatosis Increases adiponectin expression Reduces liver macrophage infiltration Reduces inflammatory adipokine mRNA	[15]
	Type 1 diabetes	Wistar rats by treatment with streptozotocin	Ameliorates Type 1 diabetes	[51]
	Type 2 diabetes	Wistar rats by treatment with nicotinamide	Increases insulin sensitivity via the PI3K-Akt-mTOR axis Suppress adipose tissue Inflammation	[50]
		Lepr−/− mice	Improves glucose tolerance Decreases fasting blood glucose Increases insulin sensitivity via Akt phosphorylation	[45]
RvD2	Obesity	Swiss mice with HFD-induced obesity	Reduces adiposity Improves glucose tolerance Increases hypothalamic expression of anti-inflammatory cytokines	[40]
		C57BL/6 mice with HFD-induced obesity	Rescues impaired expression and secretion of adiponectin Decreases pro-inflammatory adipokine production including leptin, TNF-α, IL-6 and IL-1β	[34]
	Atherosclerosis	Apoe−/− mice with HFD-induced obesity	Prevents atheroprogression Dampens macrophage-instructed inflammation	[52]
	Acute ischemic stroke with diabetes	C57BL/6 mice treatment with streptozotocin	Promotes inflammation resolution in macrophages/microglia	[53]
RvE1	Obesity	C57BL/6 mice with HFD-induced obesity	Restores fasting glucose and insulin levels	[11]
	NAFLD	ob/ob mice	Reduces liver steatosis Reduces macrophage activation Increases insulin sensitivity by inducing adiponectin, GLUT-4, IRS-1, and PPARγ expression	[54]
	Atherosclerosis	ApoE*3Leiden transgenic mice with atherogenic western-type diet	Reduces atherosclerotic lesion size Attenuates the formation of severe lesion	[55]

#### 4.1.2. E-Series Resolvins

RvE1 is a member of the E-series family of resolvins derived from EPA [55]. RvE1 has been shown to act as a potent endogenous lipid mediator, promoting anti-inflammatory activity and mitigating the pathogenic features of obesity-associated metabolic diseases [56]. Similar to D-series resolvins, RvE1 can also enhance insulin sensitivity and ameliorate glucose metabolism [11,54]. For example, RvE1 treatment of mice with HFD-induced obesity reduced hyperinsulinemia and hyperglycemia and improved fasting insulin and glucose levels [11]. Dietary administration of RvE1 to leptin-deficient obese diabetic ob/ob mice, which exhibit insulin resistance and fatty liver, increased the levels of adiponectin, a protein hormone essential for regulating glucose levels and fatty acid breakdown in adipose tissue [54]. In addition, RvE1 was found to upregulate the expression of genes involved in insulin and glucose metabolism, such as PPARγ, GLUT-4, and IRS-1, in adipose tissues and to prevent hepatic steatosis and reduce the number of macrophages in inflamed liver tissues [54]. RvE1 has also been shown to improve atherosclerosis, a thickening of the arteries that may be due to high cholesterol levels and excessive inflammation [55]. Specifically, RvE1 treatment of ApoE*3Leiden transgenic mice with atherosclerosis reduced the size of the atherosclerotic lesions and attenuated their severity by modulating the expression of the aortic genes involved in inflammatory and immune responses [55].

### 4.2. Protectin 

PDX, an isomer of protectin/neuroprotectin D1 derived from DHA, has been shown to alleviate obesity-associated inflammation and insulin resistance (Table 2). The cellular mechanisms by which PDX affects insulin resistance and inflammation have been investigated in vitro and in vivo by exploring the downstream signal transduction associated with AMPK. AMPK serves as a primary cellular energy sensor and plays a crucial role in regulating metabolic homeostasis, making it a promising therapeutic target for addressing insulin resistance and type 2 diabetes [49]. 

PDX has been found to inhibit lipid accumulation in differentiated mouse preadipocyte 3T3-L1 cells and human primary adipocytes [57] and to alleviate lipopolysaccharide (LPS)-induced adipocyte inflammation by suppressing the NF-κB pathway through AMPK activation. In addition, PDX was found to improve impaired hepatic lipid metabolism and steatosis induced by palmitate and an HFD by suppressing ER stress via AMPK-induced oxygen-regulated protein 150 (ORP150) [58]. PDX has been shown to activate AMPK in skeletal muscle, inducing the expression of interleukin (IL)-6, which attenuates insulin resistance and hepatic gluconeogenesis in diabetic mice [59]. PDX has been found to consistently enhance insulin sensitivity in muscle cells, promoting fatty acid oxidation through increased AMPK and PPARα expression [60]. Moreover, PDX was shown to ameliorate hepatic insulin resistance by modulating fetuin-A and SeP expression via the AMPK/SIRT1 pathway [61]. Taken together, these findings suggest that AMPK activation may be a mechanism responsible for the anti-inflammatory and insulin-sensitizing effects of PDX.

Although the role of PD1 in obesity-related metabolic disorders has not been fully determined, PD1 was shown to increase adiponectin expression in white adipose tissue explants from ob/ob mice [54].

**Table 2:** Effects of protectins on in vitro cell lines and in vivo animal models of obesity-induced metabolic diseases.

Metabolite	Disease	Experimental Model	Effect	Ref.
PDX	Obesity	3T3-L1 cells with LPS treatment,	Enhances insulin sensitivity by suppressing the NF-κB pathway via AMPK activation Ameliorates adipocyte Inflammation	[57]
		Human primary adipocytes with LPS treatment		
	NAFLD	HepG2 cells with palmitate treatment,	Improves hepatic lipid metabolism and steatosis through suppression of ER stress via an AMPK-ORP150 pathway	[58]
		C57BL/6 mice with HFD-induced obesity		
	Type 2 diabetes	db/db mice	Ameliorates insulin resistance Increases skeletal muscle IL-6 secretion	[59]
		C2C12 cells with palmitate treatment,	Improves insulin resistance via activation of AMPK/PPARα	[60]
		C57BL/6 mice with HFD-induced obesity		
		Human primary hepatocytes with palmitate treatment	Ameliorates hepatic insulin resistance through AMPK/SIRT1-mediated pathway	[61]
PD1	Obesity	ob/ob mice	Increases adiponectin expression	[54]

### 4.3. Maresins

MaR1, an anti-inflammatory mediator, plays a crucial role in reducing inflammation, steatosis, and fibrosis in hepatocytes [28]. Among the SPMs, MaR1 is the most validated lipid mediator for protecting against obesity-induced liver steatosis and injury (Table 3).

MaR1 treatment has been shown to reverse the effects of the pro-inflammatory cytokine TNF-α [62,63] and to enhance glucose homeostasis in obese mice [64,65]. Obesity induces hypoxia in adipose tissue, increasing oxidative stress and the production of pro-inflammatory cytokines such as TNF-α [66,67]. Elevated TNF-α levels stimulate both lipolysis [68,69] and autophagy [70] in adipose tissues. Increased lipolysis breaks down TG into fatty acids, with these excess fatty acids spilling over into the bloodstream, leading to insulin resistance. MaR1 has been shown to attenuate TNF-α-induced alterations in insulin signaling and glucose uptake [64,65], to regulate the basal expression of adipokines (e.g., adiponectin) in human adipocytes, and to counteract TNF-α-induced alterations in adipokine expression in vitro [71]. These findings suggest that MaR1 can reverse the reduction in adipokine expression in HFD-fed and TNF-α treated obese mice, potentially improving insulin sensitivity and alleviating hepatic steatosis.

MaR1 treatment has also been shown to activate AMPK and its downstream factors associated with fatty acid oxidation and autophagy [72]. This activation leads to increased AMPK phosphorylation in hepatocytes, ultimately reducing lipid accumulation and ER stress [62,73]. MaR1 has been shown to ameliorate NAFLD via AMPK/SERCA2b-mediated suppression of ER stress, while also increasing the expression of ORP150, which further inhibits ER stress [73]. Additionally, MaR1 treatment of ob/ob mice was found to activate AMPK in the white adipose tissue (WAT) [64]. Taken together, these findings suggest that AMPK plays a crucial role in MaR1-associated suppression of hepatic steatosis.

MaR1 has also been shown to alleviate NASH. The effects of MaR1 are thought to involve fibroblast growth factor-21 (FGF21), a peptide hormone primarily synthesized in the liver that regulates glucose and lipid metabolism, as well as energy homeostasis [74]. The nuclear receptor RORα has been identified as an important regulator of macrophage polarization in the liver, promoting an anti-inflammatory M2 phenotype in NASH [75]. MaR1 has been found to enhance RORα-induced M2 polarization in liver macrophages, thereby resolving liver inflammation in NASH [76]. Interestingly, RORα is activated by direct binding of MaR1 as an endogenous lipid ligand, suggesting that the MaR1–RORα axis may link inflammation and metabolic dysfunction in the liver. Overall, the ability of MaR1 to ameliorate NAFLD-related injuries offers a promising therapeutic avenue to prevent the progression from simple steatosis to NASH in patients with fatty liver disease.

**Table 3:** Effects of maresins on in vitro cell lines and in vivo animal models of obesity-induced metabolic diseases.

Metabolite	Disease	Experimental Model	Effect	Ref.
Mar1	Obesity	3T3-L1 cells	Ameliorates TNF-α-induced alterations on lipolysis and autophagy	[63]
		Primary human adipocytes,	Modulates adipokine expression	[71]
		C57BL/6 mice with HFD-induced obesity		
	Obesity; Type 2 diabetes	ob/ob and C57BL/6 mice with HFD-induced obesity	Improves the insulin tolerance Inhibits the activity of TNF-α Increases adiponectin secretion Increases Akt and AMPK activation	[64]
		hMSC-derived adipocytes, C57BL/6 mice with HFD-induced obesity	Improves the insulin tolerance Inhibits the activity of TNF-α Promotes Akt signaling	[65]
	NAFLD	C57BL/6 mice with HFD-induced obesity	Ameliorates hepatic steatosis and lipotoxic ER stress via AMPK activation	[62]
		Mouse primary hepatocytes with palmitate treatment,	Attenuates hepatic steatosis Suppresses ER stress via AMPK/SERCA2b pathway	[73]
		C57BL/6 mice with HFD-induced obesity		
		ob/ob and C57BL/6 mice with HFD-induced obesity	Ameliorates hepatic triglycerides and fasting glucose levels Increases fatty acid oxidation genes and autophagy via AMPK activation	[72]
	NASH	Fat-1 transgenic mice with HFD-induced obesity	Ameliorates hepatic steatosis and fibrosis Enhances the M2 polarity of liver macrophages	[76]

## 5. The Therapeutic Potential of SPMs in the Clinical Treatment of Lipid Metabolic Disorders

**Figure 3:**
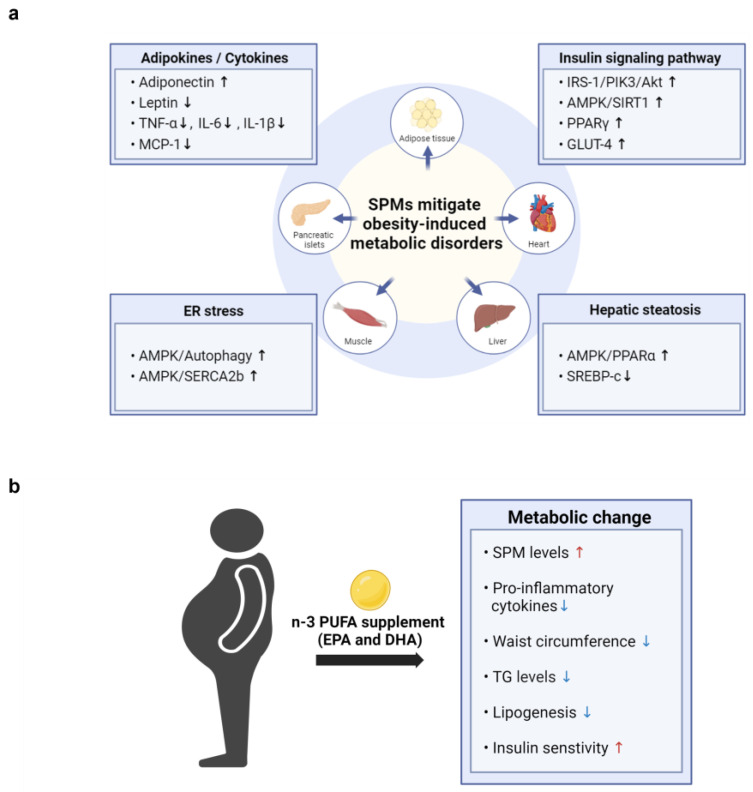
The potential cellular mechanisms and metabolic effects of SPMs in mitigating obesity-induced metabolic disorders. (**a**) SPMs mitigate obesity-induced metabolic disorders by influencing key cellular mechanisms across various tissues, including adipocytes, heart, liver, muscle, and pancreatic islets. These mechanisms involve the modulation of adipokine and cytokine levels, enhancement of insulin signaling pathways, reduction in ER stress, and alleviation of hepatic steatosis. (**b**)The figure illustrates the effects of n-3 PUFA supplementation on obesity-related parameters observed in obese patients. Supplementation with n-3 PUFAs increases SPM levels and insulin sensitivity, while decreasing waist circumference, lipogenesis, and the levels of triglycerides (TG) and pro-inflammatory cytokines.

Several studies have demonstrated that SPMs and their metabolic intermediates can reduce inflammation and enhance insulin sensitivity in animal models of obesity, thereby improving a range of obesity-related complications. As illustrated in Figure 3a, SPMs mitigate obesity-induced metabolic disorders across various tissues by regulating adipokine and cytokine levels, enhancing insulin signaling pathways, reducing ER stress, and alleviating hepatic steatosis (Table 1, Table 2 and Table 3). To date, however, clinical trials have not assessed the efficacy of specific SPMs in treating obesity-associated metabolic disorders.

Most studies to date have investigated the effects of dietary supplementation with fish oil, n-3 fatty acids, or EPA and DHA, rather than specific types of SPMs, on metabolic parameters in obese individuals (Figure 3b). In a recent nonrandomized, uncontrolled clinical trial, adults with obesity took 2 g/day of a marine oil-derived n-3 PUFA supplement for 4 weeks to evaluate its effect on circulating levels of SPMs. Six SPM precursors and two important SPMs, RvE1 (3.5-fold) and MaR1 (4.7-fold), were increased following supplementation, indicating that n-3 PUFA supplementation can significantly boost the levels of specific SPMs in the bloodstream of both healthy individuals and those with obesity [77].

Moreover, n-3 PUFA supplementation has shown promising effects on various metabolic parameters in individuals with obesity-related metabolic disorders. For example, n-3 PUFA administration was found to improve waist-to-height ratio, waist circumference, and blood concentrations of TG, glucose, and glycosylated hemoglobin in patients with type 2 diabetes [78,79]. Moreover, n-3 PUFA supplementation decreased fasting insulin levels in individuals with hyperinsulinemia [80], as well as being associated with decreased lipogenesis and liver steatosis [81]. In addition, n-3 PUFAs were shown to modulate the expression of genes associated with lipid accumulation and collagen deposition while reducing inflammatory macrophages in adipose tissue from individuals with insulin resistance [82]. Collectively, these findings suggest a potential role for n-3 PUFAs in improving metabolic health across various contexts.

Although n-3 PUFAs have shown potential in modulating key risk factors of obesity-related metabolic disorders, including TG, glucose levels, and inflammation (Figure 3), clinical studies assessing the effects of dietary supplementation with n-3 PUFAs on metabolic parameters in obesity have yielded conflicting results. For example, n-3 PUFAs were reported to have neutral effects on metabolic profiles in patients with type 2 diabetes [83,84]. Discrepancies among studies may be attributed to various factors such as sample sizes, baseline patient characteristics, fatty acid doses and purity, length of treatment, and concomitant medications.

Collectively, these findings indicate that supplementation with SPMs has promising therapeutic potential in patients with metabolic disorders. However, it is important to recognize the unpredictable enzymatic pathways involved in producing n-3 derivatives during PUFA supplementation. A deeper understanding of the mechanisms by which these factors influence the incorporation of PUFAs into the lipid pool, along with their metabolism and downstream molecular effects, will enable a more effective design of n-3 PUFA supplementation strategies in the treatment of obesity. Additionally, a better understanding of the factors contributing to variability in the clinical effects of n-3 PUFAs may help tailor supplementation regimens to achieve greater benefits for individual patients. Future research therefore requires careful consideration of study design and patient characteristics.

## 6. Discussion

In summary, this review of the clinical applications and therapeutic benefits of SPMs in patients with obesity-related metabolic diseases revealed promising avenues for intervention. Obesity-associated inflammation is closely linked to reduced SPM levels, prolonging unresolved inflammation and potentially worsening metabolic disorders. Supplementation with n-3 PUFAs enhances the levels of SPM precursors and SPM, effectively reducing inflammation and alleviating obesity. In addition, certain SPMs were found effective in attenuating hepatic inflammation and steatosis in experimental models of obesity, highlighting the pivotal roles of these SPMs in managing obesity-related metabolic diseases. The significant anti-inflammatory and pro-resolving properties of SPMs suggest their possible use as potential therapeutic agents in the treatment of obesity-related conditions. Furthermore, the ability of these SPMs to modulate macrophage polarization and resolve inflammation suggests that they may play a promising role in therapeutic interventions.

Since the effects of SPMs are primarily studied in animal models or in vitro cell systems, it is crucial to account for species differences and variations in experimental designs. Given that SPM profiles can vary between animal models and humans, findings from animal studies might not always translate directly to humans. This underscores the need for careful interpretation of preclinical results and highlights the importance of validating SPMs in human studies, especially regarding obesity-induced metabolic syndrome. Researchers should aim to bridge the gap between animal models and human applications by either identifying SPMs with consistent effects across species or by improving animal models to better replicate human conditions.

Transcriptome analysis is also essential to determine the mechanisms by which obesity diminishes SPM levels and sensitivity. Identifying key changes in the signaling pathways associated with SPM supplementation in experimental models of obesity could reveal new treatment avenues and enhance the monitoring of disease progression and patient prognosis. Furthermore, integrative analyses combining metabolite and transcriptome data are crucial for elucidating the detailed molecular mechanisms of SPM action. These studies may shed light on changes in endogenous oxylipin levels following SPM treatment and the ability of SPMs to regulate gene expression, providing critical insights into managing obesity and the related metabolic diseases. 

Understanding these mechanisms is crucial for optimizing SPM supplementation and identifying the precise molecular and cellular pathways involved in inflammatory pathologies. This approach may not only advance the development of effective clinical applications but also reveal novel metabolic pathways associated with the progression or resolution of obesity-related metabolic diseases. In turn, this may enable innovative therapeutic strategies and the identification of noninvasive diagnostic biomarkers for metabolic disorders.
